# Enhancing understanding of SARS-CoV-2 infection among individuals with Down syndrome: An integrative review

**DOI:** 10.1590/1516-3180.2023.0015.R1.230523

**Published:** 2023-08-21

**Authors:** Maria Vitoria Gomes da Silva, Laura Resende Guimarães Pereira, Lucimar Retto da Silva de Avó, Carla Maria Ramos Germano, Débora Gusmão Melo

**Affiliations:** I Medical Undergraduate Student, Department of Medicine, Universidade Federal de São Carlos (UFSCar), São Carlos (SP), Brazil.; II Medical Undergraduate Student, Department of Medicine, Universidade Federal de São Carlos (UFSCar), São Carlos (SP), Brazil.; III MD, PhD. Associate Professor, Department of Medicine, Universidade Federal de São Carlos (UFSCar), São Carlos (SP), Brazil.; IV MD, PhD. Associate Professor, Department of Medicine, Universidade Federal de São Carlos (UFSCar), São Carlos (SP), Brazil.; V MD, PhD. Full Professor, Department of Medicine, Universidade Federal de São Carlos (UFSCar), São Carlos (SP), Brazil.

**Keywords:** COVID-19, SARS-CoV-2, Down syndrome, Systematic review [publication type], Trisomy 21, Down syndrome comorbidities, Coronavirus disease 2019, Cytokine storm in coronavirus disease 2019, Outcomes of coronavirus disease 2019 in individuals with Down syndrome, Integrative review

## Abstract

**BACKGROUND:**

Down syndrome (DS) is a non-rare genetic condition that affects approximately 1 in every 800 live births worldwide. Further, it is associated with comorbidities, anatomical alterations of the respiratory tract, and immunological dysfunctions that make individuals more susceptible to respiratory infections.

**OBJECTIVE:**

To systematize the current scientific knowledge about the severe acute respiratory syndrome coronavirus 2 (SARS-CoV-2) infection among individuals with DS.

**DESIGN AND SETTING:**

This integrative review was conducted at the Universidade Federal de São Carlos, São Paulo, Brazil.

**METHODS:**

This review was conducted in the following databases: the Virtual Health Library (Biblioteca Virtual em Saúde, BVS), PubMed, and Web of Science, using MeSH descriptors. The search included English or Portuguese studies published between January 1, 2020, and October 14, 2022.

**RESULTS:**

A total of 55 articles from 24 countries were selected, comprising 21 case-control or cohort studies, 23 case reports or series, and 11 narrative reviews or opinion studies. The articles were grouped into five categories: previous comorbidities, coronavirus disease 2019 (COVID-19) clinical features and evolution, cytokine storm and interleukins, living in institutions as a risk factor, and behavioral actions as a protective factor against SARS-CoV-2 infection.

**CONCLUSION:**

Individuals with DS are more susceptible to COVID-19 infection due to variables such as previous comorbidities, immunological factors, and their habitable environments. These aspects confer a higher risk of infection and an unfavorable clinical course. The precise pathways involved in the pathophysiology of COVID-19 in individuals with DS are not clear, thus requiring further studies.

**SYSTEMATIC REVIEW REGISTRATION:**

The Open Science Framework registered the research protocol (https://osf.io/jyb97/).

## INTRODUCTION

Down syndrome (DS) is a non-rare genetic condition that affects approximately 1 in every 800 live births worldwide.^1^ Phenotypically, DS is characterized by intellectual and developmental disabilities, facial dysmorphisms, muscular hypotonia, and numerous birth defects, including cardiac and gastrointestinal anomalies.^1,2^ Furthermore, individuals with DS have several immune defects, making them more susceptible to autoimmune diseases and infections, especially respiratory tract infections, which represent a relevant cause of mortality.^3–5^

The immune dysregulation in DS results from various factors spanning innate and adaptive systems.^5^ There is a decrease in the number of natural killer cells, monocytes, and dendritic cells, in addition to decreased neutrophil chemotaxis. Moreover, thymus hypoplasia leads to significantly reduced T-lymphocyte numbers.^5^ There is also a reduced number of all B-cell populations, especially switched memory B cells, which impair the adaptive immune response.^5,6^ Additionally, structural alterations in the respiratory system, such as tracheomalacia and laryngomalacia, make it challenging to remove mucus and facilitate the colonization of the respiratory tract by pathogens.^5^

The life expectancy of individuals with DS has increased over the last few decades; nowadays, it exceeds 60 years.^4^ In early childhood, congenital heart defects are the principal cause of death while in other stages of life, respiratory infections are the most common. Apart from respiratory diseases, neurological disorders such as dementia represent a risk for mortality in middle age.^1,4^

The SARS-CoV-2 infection has different courses in individuals with DS depending on comorbidities, changes in the immune response to the virus, time of infection, and therapeutic approaches.^7^ The lack of systematized information on how the disease affects this population is a barrier to discussing the specific risk of coronavirus disease 2019 (COVID-19).^8^

## OBJECTIVE

This integrative review aimed to systematize the current scientific knowledge about the behavior of severe acute respiratory syndrome coronavirus 2 (SARS-CoV-2) infection among individuals with DS. In particular, to enhance understanding of the subject and identify gaps in the area.

## METHODS

### Research design

This integrative literature review was conducted per the literature^9,10,11^ based on the Preferred Reporting Items for Systematic reviews and Meta-Analyses statement (PRISMA).^12^ The research protocol was registered in the Open Science Framework (https://osf.io/jyb97/).^13^

Six steps were followed to ensure methodological rigor: (1) elaboration of the research question, selection of the databases, and identification of the descriptors; (2) definition of inclusion and exclusion criteria and search in databases; (3) data extraction from selected studies; (4) critical analysis of the included studies; (5) interpretation and discussion of the data; and (6) presentation of acquired knowledge.^14^

The guiding question of this review was: How is the infection behavior of the SARS-CoV-2 in individuals with DS? This question was designed by the population, intervention, comparison, outcome (PICO) strategy, as detailed in Table 1.^15^

**Table 1.** Research question following PICO parameters

P	Population	Who was studied?	Individuals with Down syndrome
**I**	Intervention	What happened?	Infection by SARS-CoV-2
**C**	Comparison	Comparison between populations	Individuals without Down syndrome
**O**	Outcome	What is the prognostic?	Down syndrome influences clinical infection caused by COVID-19, assessed by outcomes such as infection rates, morbidity, hospital admission, ICU stay, duration of hospital stay, mortality, complications, sequelae, etc.

COVID-19 = coronavirus disease 2019; SARS-CoV-2 = severe acute respiratory syndrome coronavirus 2; ICU = intensive care unit.

### Search strategy

Literature searches were conducted in three databases: the Virtual Health Library (*Biblioteca Virtual em Saúde*, BVS), PubMed, and Web of Science. In the BVS, Latin American and Caribbean Literature on Health Sciences (LILACS) databases were accessed, providing access to the Scientific Electronic Library Online (SciELO) database and the Pan American Health Organization Institutional Repository Information Sharing (PAHO-IRIS) database. In PubMed, the MEDLINE database was accessed and in the Web of Science database, the core collection was accessed.

For the search, we defined the following descriptors from the Medical Subject Headings (MeSH): ((47,XX,+21) OR (47,XY,+21) OR (Down Syndrome, Partial Trisomy 21) OR (Down’s Syndrome) OR (Mongolism) OR (Partial Trisomy 21 Down Syndrome) OR (Trisomy 21) OR (Trisomy 21, Meiotic Nondisjunction) OR (Trisomy 21, Mitotic Nondisjunction) OR (Trisomy G)) AND ((SARS-CoV-2) OR (SARS-CoV-2 Virus) OR (SARS-CoV-2 Infection) OR (COVID-19) OR (COVID-19 Virus) OR (COVID19) OR (COVID-19 Pandemic) OR (COVID-19 Pandemics) OR (COVID-19 Virus Disease) OR (COVID-19 Virus Infection) OR (2019 Novel Coronavirus) OR (2019 Novel Coronavirus Disease) OR (2019 Novel Coronavirus Infection) OR (2019-nCoV) OR (2019-nCoV Disease) OR (2019-nCoV Infection) OR (Coronavirus Disease 2019) OR (Coronavirus Disease-19) OR (Coronavirus Disease 2019 Virus) OR (SARS Coronavirus 2 Infection) OR (SARS Coronavirus 2)) AND ((Cytokines) OR (Cytokine) OR (Pneumonia, Viral) OR (Risk Factors) OR (Health Correlates) OR (Population at Risk) OR (Populations at Risk) OR (Comorbidity)).

Two independent authors performed the searches on the databases. The compatibility of the material found was checked and then entered into the Rayyan software (Cambridge, United States, https://www.rayyan.ai/). Duplicate studies were identified and excluded using Rayyan. Title and abstract screening were applied to identify relevant studies in blind mode by two reviewers. When there was disagreement or doubt, a third reviewer was consulted. Finally, the articles were selected after a consensus discussion. The selected studies were read in their entirety. Further, with the help of the eligibility criteria, they were included or excluded from this review.

### Eligibility criteria

This review included papers published in English or Portuguese between January 1, 2020, and October 14, 2022. Manuscripts that discussed the infection of SARS-CoV-2 in individuals with DS, regardless of the methodology and type of study, were included. The exclusion criteria were: articles without adherence to the theme; not involving humans; in different languages; and duplicated texts in the databases.

### Data extraction and quality assessment

Data from the selected studies were extracted using a form (Supplemental file available at https://doi.org/10.6084/m9.figshare.21277452.v4),^16^ which made it possible to summarize the information, verify the validity of the studies, and identify the relationships in the data. The following information was collected: authors, country of origin or year of publication, journal, study method, main results, and study conclusions.

The quality of the studies was evaluated and categorized by the level of evidence using the following criteria, modified from Melnyk and Fineout-Overholt:^17^ I – a systematic review with meta-analysis of randomized controlled trials; II – randomized controlled trial; III – non-randomized controlled trial; IV – case-control or cohort study; V – a systematic review of descriptive or qualitative studies; VI – descriptive or qualitative study (including case reports and case series); VII – narrative review or expert opinion.

The authors reviewed the final studies independently and then worked collaboratively to establish the discussed categories. This inductive categorization allowed us to identify the main themes from the articles’ results.^18^

## RESULTS

**Figure 1.**
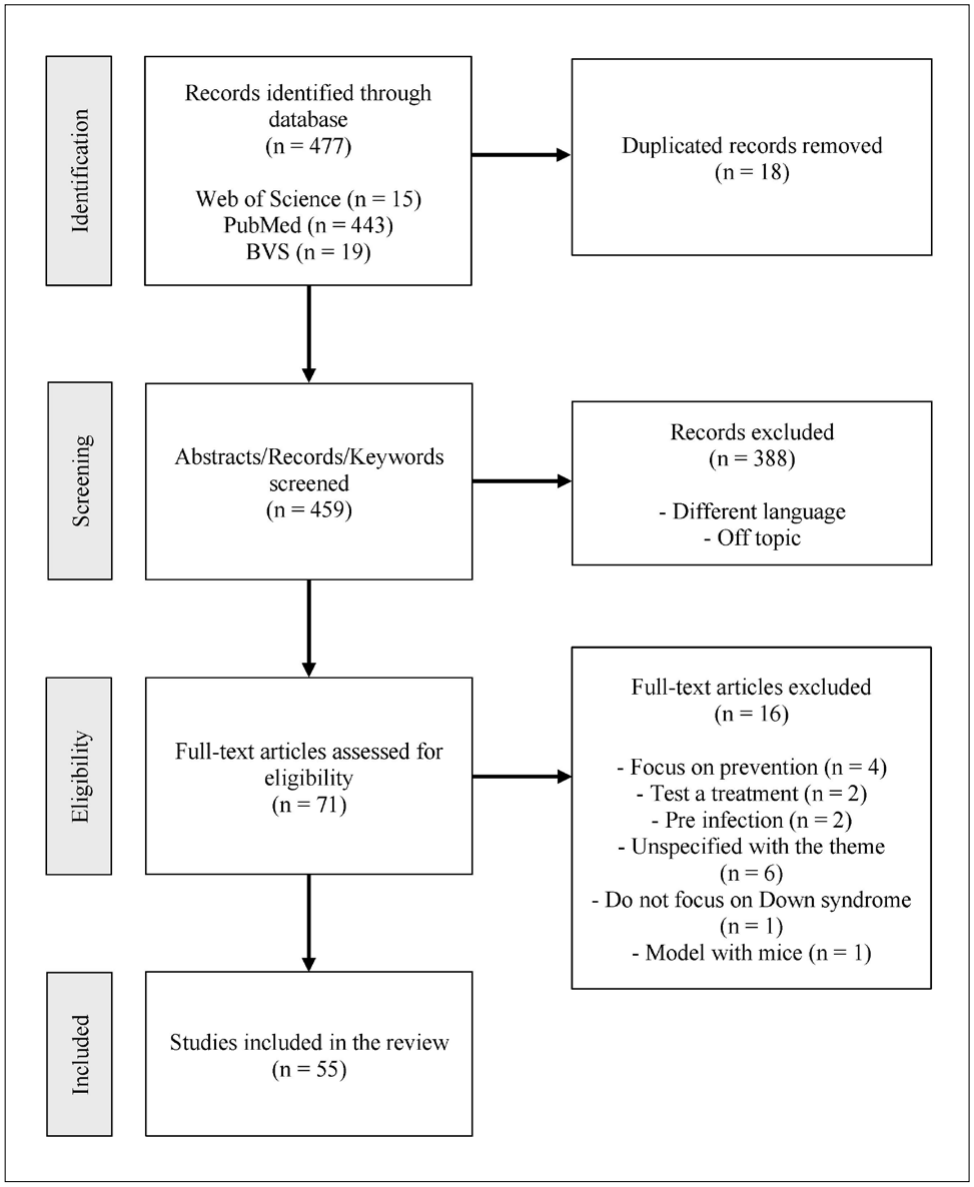
The flow of the selection process of articles of this integrative review.

A total of 477 studies were identified in the databases in the initial search. After applying the eligibility criteria, 55 manuscripts were selected for this review. The selection process and exclusion reasons are described in Figure1. The articles included were named A1 through A55.

The selected articles are described in Table 2, which summarizes their location of origin, type of study, and level of evidence. The studies were conducted in 24 different countries. The United States of America (USA) comprises the highest number of studies (n = 16), followed by Brazil (n = 9), Spain (n = 8), and Italy (n = 6). We did not find studies with evidence levels I, II, III, or V. All 55 selected studies consisted of a case report or case series, cohort, case-control, review, or expert opinion with evidence levels IV, VI, and VII, respectively.

**Table 2.** Characterization and evidence level of studies included in this review

Article	Authors/Year	Location	Type of study	Evidence level	Number of individuals with DS and COVID-19
A1	Malle et al. (2021)^19^	USA	Case-control	IV	12
A2	El Kaouini et al. (2021)^30^	Morocco	Case report	VI	2
A3	De Cauwer and Spaepen (2021)^65^	Belgium	Case series	VI	4
A4	Dard, Janel and Vialard (2020)^70^	France	Expert opinion	VII	0
A5	Wadman (2020)^71^	USA	Expert opinion	VII	1
A6	Del Carmen et al. (2020)^75^	Spain	Expert opinion	VII	0
A7	Kantar et al. (2020)^36^	Italy	Case report	VI	2
A8	Real de Asua et al. (2021)^37^	Spain	Cohort	IV	86
A9	Russo et al. (2020)^74^	Brazil	Expert opinion	VII	0
A10	Babamahmoodi et al. (2020)^66^	Iran	Case report	VI	2
A11	Villani et al. (2020)^38^	Italy	Case series	VI	16
A12	Robayo et al. (2021)^39^	Colombia	Case report	VI	1
A13	Khoshnood et al. (2021)^40^	USA	Case report	VI	1
A14	Vita et al. (2021)^7^	Italy	Case report	VI	2
A15	Altable and de la Serna (2021)^67^	Spain	Narrative review	VII	0
A16	Kim-Hellmuth et al. (2021)^41^	Germany	Case report	VI	1
A17	Krishnan et al. (2020)^42^	USA	Case series	VI	3
A18	Emami et al. (2021)^20^	Iran	Case-control	IV	18
A19	Clift et al. (2021)^21^	UK	Cohort	IV	4,053
A20	Newman et al. (2021)^22^	USA	Case series	VI	4
A21	Stefanuto et al. (2021)^23^	Brazil	Case report	VI	1
A22	Simpson et al. (2020)^24^	Georgia	Case series	VI	3
A23	Perera et al. (2020)^25^	England and Ireland	Case series	VI	20
A24	Oyanagi et al. (2021)^26^	Japan	Case report	VI	1
A25	Malle et al. (2021)^27^	USA and Spain	Case report	VI	2
A26	Huls et al. (2021)^28^	USA, UK, Brazil, Italy, Spain, France, India	Case-control	IV	1,046
A27	Alsahabi et al. (2021)^29^	Saudi Arabia	Case report	VI	1
A28	Landes et al. (2021)^72^	USA	Cohort	IV	20
A29	De Toma and Dierssen (2021)^53^	Spain	Narrative review	VII	0
A30	Hippisley-Cox et al. (2021)^63^	England	Cohort	IV	3,963*
A31	Williamson et al. (2021)^55^	England	Cohort	IV	341
A32	Semenzato et al. (2021)^56^	France	Cohort	IV	256
A33	Santos et al. (2020)^64^	Brazil	Cohort	IV	73
A34	Bergman et al. (2021)^57^	Sweden	Case-control	IV	85
A35	Illouz et al. (2021)^31^	Israel	Cohort	IV and VI	20
A36	Illouz et al. (2021)^68^	Israel, USA, Spain, Canada, Switzerland.	Narrative review	VII	0
A37	Espinosa (2020)^51^	USA	Narrative review	VII	0
A38	Ma et al. (2021)^49^	USA	Case report	VI	1
A39	Amin et al. (2022)^32^	Bangladesh	Case report	VI	1
A40	Baksh et al. (2022)^33^	UK	Cohort	IV	651
A41	Boschiero (2022)^58^	Brazil	Cohort	IV	5,152
A42	Emes et al. (2021)^46^	USA, UK, Brazil, Italy, Spain, France, India, Germany	Cohort	IV	328
A43	Evangelho et al. (2022)^54^	Brazil	Expert opinion	VII	0
A44	Kobayashi et al. (2022)^43^	Japan	Case report	VI	1
A45	Koyama et al. (2022)^62^	USA	Cohort	IV	1,412
A46	Ku et al. (2022)^59^	USA	Cohort	IV	142
A47	Kuczborska, Buda and Ksiazyk (2022)^44^	Poland	Case Report	VI	1
A48	Pinku et al. (2022)^34^	UK, India	Cohort	IV	1,272
A49	Shi et al. (2022)^60^	Scotland	Cohort	IV	79
A50	Silva et al. (2022)^35^	Brazil	Case series	VI	3
A51	Lunsky et al. (2022)^61^	Canada	Cohort	IV	121
A52	Majithia and Ribeiro (2022)^50^	USA	Expert opinion	VII	0
A53	Magalhães et al. (2022)^45^	Brazil	Case-control	IV	7
A54	Parasini et al. (2022)^52^	Italy	Case series	VI	6
A55	Atkinson et al. (2022)^69^	USA	Expert opinion	VII	0

DS = Down syndrome; COVID-19 = coronavirus disease 2019; UK = United Kingdom; USA = United States of America. * The number refers to all individuals with DS in the study, not just those with COVID-19.

We organized these 55 manuscripts per the similarity of data and themes, grouping them into five categories as reported in Table 3. The categories represent elementary DS-related issues and changes resulting from the SARS-CoV-2 infection. Some studies were included in more than one category.

**Table 3.** Characterization of categories and studies included in each of them

Categories	Description of the category	Articles
1. Previous comorbidities in individuals with DS	It connects previous comorbidities in individuals with DS, such as seizures, dementia, heart defects, obesity, hypothyroidism, and apnea, with the SARS-CoV-2 infection.	A1, A2, A7, A8, A11, A12, A13, A14, A16, A17, A18, A19, A20, A21, A22, A23, A24, A25, A26, A27, A35, A39, A40, A42, A44, A47, A48, A50, A53.
2. Clinical features and evolution of SARS-CoV-2 infection in individuals with DS	It identifies symptoms related to COVID-19 in individuals with DS, the natural history of the disease, the occurrence of coinfections, and the outcomes in these individuals.	A1, A7, A11, A12, A13, A18, A19, A20, A21, A22, A24, A26, A29, A30, A31, A32, A33, A34, A37, A38, A39, A41, A43, A44, A45, A46, A49, A50, A51, A52, A54.
3. Cytokine storm, interleukins, and other laboratory changes	It identifies immunological dysfunctions in individuals with DS and COVID-19.	A2, A3, A10, A12, A15, A16, A25, A29, A36, A37, A39, A47, A50, A52, A55.
4. Living in institutions as a risk factor	It addresses the more severe course of SARS-CoV-2 infection in individuals with DS living in institutions.	A4, A5, A23, A28, A40.
5. Behavioral actions as a protective factor against SARS-CoV-2 infection	It shows that specific behavioral patterns in individuals with DS may be a protective factor against infection by SARS-CoV-2.	A6, A9.

SARS-CoV-2 = severe acute respiratory syndrome coronavirus 2; DS = Down syndrome; COVID-19 = coronavirus disease 2019.

## DISCUSSION

Different features, such as previous biological features, interactions with the environment, and behavior patterns, have been described as modifiers of risk and outcome for SARS-CoV-2 infection in patients with DS.

### First category: Previous comorbidities in individuals with Down syndrome

Six main comorbidities directly or indirectly interfered with the clinical course of SARS-CoV-2 infection in individuals with DS: dementia, epilepsy, heart defects, sleep apnea, obesity, and thyroid pathologies.^7,19–42^

Higher dementia rates were observed in individuals with DS and COVID-19, when compared with individuals with COVID-19 without DS^19,21,38^ and individuals with DS and respiratory infections caused by other etiologies.^37^ Illouz et al.^31^ described a change in the endocytosis process in individuals with DS related to some genes located on chromosome 21, including *Amyloid Beta Precursor Protein (APP)*, known to mediate dementia in these individuals. This gene is also involved in viral trafficking, changing endosomal fusion, which may be one factor that favors a higher risk of COVID-19 in individuals with DS and dementia.^31^

Epilepsy was a relevant comorbidity in individuals with intellectual disability and COVID-19,^21^ and it was more common in individuals with DS.^7,19,25,30,37^ It was indirectly associated with an unfavorable outcome related to other comorbidities and care challenges.

Heart defects were recurrent in DS individuals with COVID-19, mainly in the pediatric age group.^20–24,26–29,35,36,40–45^ Children with DS seem more likely to be exposed to severe COVID-19 than those without DS.^46^

Simpson et al.^24^ presented a case series of seven children, three of whom had DS, a heart defect, and COVID-19. In addition to the corrected tetralogy of Fallot, one of these infants had hypothyroidism and obstructive sleep apnea and died 2.5 months after a SARS-CoV-2 infection.^24^ The other cardiopathies mentioned were primarily septal defects that were associated with severe infection and prolonged hospitalization.^24,26,27,36,40–42,44^ Some studies stated whether the heart defect was surgically corrected, while others did not, making it difficult to conclude if surgical treatment of the heart disease changes the natural progression or outcome of the COVID-19 disease.

Sleep apnea was a comorbidity associated with obesity and heart disease and was also prevalent in the pediatric age group.^22,24,36,40,42^ Apnea has been linked to a more severe course of COVID-19, ventilatory support, prolonged hospitalization, and death.^24,36,42^

Hypothyroidism was persistent;^7,19,24,32,35,38,39^ Malle et al.^19^ demonstrated a 50% prevalence among patients with DS hospitalized due to COVID-19. Despite this, thyroid disease did not appear to have played a direct role in the progression of COVID-19.^19^

Primary data revealed a worse prognosis in patients with more than one comorbidity. The cohort study by Pinku et al.^34^ suggested that individuals with DS from low-income countries may have more comorbidities due to structural socioeconomic inequality.

Among the comorbidities, dementia plays an important role, due to its frequent association with other disorders. Interestingly, dementia and epilepsy had already been associated with complications of recurrent infections and premature death even before the COVID-19 pandemic.^47^ In a cross-sectional study comprising 878 adults with DS over 45 years old, Bayen et al.^48^ reported a 40% prevalence of dementia. It revealed that individuals with DS and dementia had more comorbidities than those without dementia and younger individuals. In particular, four treatable conditions – hypothyroidism, epilepsy, anemia, and weight loss - were more frequent in individuals with DS and dementia.^48^

### Second category: Clinical features and evolution of SARS-CoV-2 infection in individuals with Down syndrome

Although the main clinical manifestations of COVID-19 infection in individuals with DS are similar to those in other individuals, i.e., respiratory distress, fever, cough, and muscle pain,^20^ the literature suggests that individuals with DS may have a distinct initial clinical presentation of COVID-19,^22,26,38,39,43,49,50^ showing atypical symptoms such as hemoptysis, vomiting, diarrhea, abdominal pain, and autoimmune manifestations.^22,26,38,43,49^ Additionally, unusual symptoms such as arrhythmia can be caused by underlying pathologies and mask the presence of respiratory symptoms.^24,26^ These unusual symptoms were not directly related to a more severe course of COVID-19. Nonetheless, it can be associated with a delay in diagnosis and treatment, potentially resulting in worse outcomes.

Callea et al.^8^ created a series of health education activities for individuals with DS and their families, emphasizing the importance of recognizing typical and atypical symptoms and notifying suspected cases to the health team. Furthermore, the same group developed protocols for healthcare professionals, with guidance on testing and managing COVID-19 in individuals with trisomy 21.^8^

One study described SARS-CoV-2 and tuberculosis coinfections,^23^ in which the latter was diagnosed during hospitalization despite the individual having classic symptoms and having been in contact with a sibling already treated for tuberculosis. Furthermore, bacterial coinfections were prevalent complications in individuals with DS and COVID-19 and were described as the leading cause of death.^21,28,35,38,51,52^ Through genetic bioinformatics analysis, De Toma and Dierssen^53^ mapped the transcriptomic changes induced by trisomy 21 in pathways and proteins known to be affected by SARS-CoV-2, identifying risk factors for COVID-19 at different stages of infection.^53^ The presence of the tripled *transmembrane protease serine 2* gene *(TMPRSS2)*, located on chromosome 21, including an elevation of the bradykinin B1 receptor during the initial phase of the viral invasion, is related to angiotensin converting enzyme 2 (ACE-2). ACE-2 binds the viral protein S, facilitating viral entry into the host cell. This predisposes individuals with DS to severe acute respiratory syndrome.^53,54^ Subsequently, in the immunopathogenesis of the disease, the authors detected negative regulation of the *NLR family pyrin domain containing 3* gene *(NLRP3)*, which is involved in the immune system and is critical in maintaining homeostasis against infections. This would hypothetically contribute to the co-occurrence of viral and bacterial infections.^53^

Individuals with DS were four times more likely than the general population to acquire the SARS-CoV-2 infection.^21^ They had a more severe clinical course and required more hospitalization^52,55–61^ and intensive care unit (ICU) treatments.^28,58,61,62^ Hüls et al.,^28^ in a multicenter retrospective study involving 1,046 individuals with DS and COVID-19, showed a hospitalization rate of 56%, in which 50% were in the ICU. In a sample comprising 12 individuals with DS, Malle et al.^19^ described sepsis in 10 patients (83%). Post-infectious conditions such as Kawasaki disease^36^ and multisystem inflammation syndrome (MIS-C)^40^ were observed in two pediatric patients. SARS-CoV-2 infection severity may be related to long COVID-19 or post-COVID conditions.^32,50^

The risk of mortality was also higher.^21,56,59,61,63,64^ In the cohort study by Semenzato et al.,^56^ involving 87,809 individuals hospitalized for COVID-19 and 256 individuals with DS, the chromosomal condition was the main factor associated with the risk of hospitalization and hospital mortality, ahead of five other comorbidities: intellectual disability, lung transplantation, kidney transplantation, end-stage renal disease, and lung cancer.^56^ Similarly, in a cohort study carried out with the Brazilian population involving 73 individuals with DS and COVID-19 who died, Santos et al.^64^ reported a 32% drop in the survival rate between five and 10 days of hospitalization, demonstrating that trisomy and length of hospital stay impacted the mortality. In a cohort study involving 4,053 patients with DS and COVID-19, Clift et al.^21^ estimated a mortality rate 10 times greater than that of the general population. Hippisley-Cox et al.^63^ showed a 12.7-fold more significant risk of death in those with trisomy in a sample of vaccinated individuals that included 3,963 individuals with DS.

It is essential to consider that most of the literature data come from hospital statistics. Individuals with a milder clinical course who remain at home during the infection are frequently overlooked. Consequently, the community’s epidemiological picture of the illness is not fully covered.

### Third category: Cytokine storm, interleukins, and other laboratory changes

Elevated levels of inflammatory markers such as interleukin (IL)-6, IL-8, interferon (IFN), tumor necrosis factor (TNF), C-reactive protein, D-dimer, and lactate dehydrogenase were found in individuals with DS and COVID-19.^27,30,35,39,41,44,51,65–67^ Individuals with DS tended to have a higher early initial response to infection, especially through the action of IFNs, which could theoretically contain the viral spread. However, it is known that the coronavirus family, in general, has developed strategies to evade the effects of IFN, which probably also occurred with SARS-CoV-2.^32,51,68^

During the illness, individuals with DS often experience a cytokine storm influenced by viral action. De Toma and Dierssen^53^ described the elevation of chemokines, specifically CXCL10, which, through stimulation of monocytes and IL-10, recruits fibrocytes and aids in the activation of macrophages, facilitating lung damage such as fibrosis and leads to a more severe manifestation of the disease. As chromosome 21 encodes four of the six types of interferon receptors, an extra copy of this chromosome can result in higher plasma levels of interferons.^32,50^

Altable and de la Serna^67^ reported that changes in the levels of pro-inflammatory cytokines in individuals with DS could fluctuate with age, directly influencing the exacerbation of the immune response. There would be an immunodeficiency during childhood, resulting in a diminished pro-inflammatory response with lower levels of pro-inflammatory cytokines such as IL-2. Conversely, adults would experience the opposite scenario, with higher IL-2, IL-6, and TNF-α levels amplifying the inflammatory response. In both profiles, unfavorable factors seem to be associated with DS individuals: in childhood, they are more vulnerable to infection owing to immunodeficiency. However, in the adult stage, once infected, the increased immune response predisposes them to a worse prognosis.

Babamahmoodi et al.^66^ reported two cases of adult individuals with DS and COVID-19: one patient had higher levels of IL-6 and died in three days due to respiratory failure, while another patient had lower IL-6 values and, despite developing severe COVID-19, had a favorable outcome.

High levels of inflammation seem to predispose to more severe disease and affect vaccine response in these individuals, proving to be a risk factor in the COVID-19 protection of individuals with DS and requiring greater attention to the doses and security measures.^50,69^

### Fourth category: Living in institutions as a risk factor

Considering that some individuals with DS live in communities or institutions, this population has been assigned as a COVID-19 risk group due to the ease of viral transmission.^25,33,70,71^ More populated places seem to be associated with higher infection rates.^70^ In a cohort of 543 individuals with intellectual disabilities, 56 of whom had DS, the trisomy of 21 represented a higher risk for COVID-19 infection.^72^

The demographic profile of institutionalized individuals with DS has been described as slightly different from that of individuals with intellectual disabilities due to other etiologies. Individuals with trisomy are usually younger and more likely to have dementia, hearing loss, or be overweight.^73^ These variables might be linked to a higher vulnerability to COVID-19.

### Fifth category: Behavioral actions as a protective factor against SARS-CoV-2 infection

Since the COVID-19 pandemic started, behavioral actions have been a vital tool to prevent infection by SARS-CoV-2. In this context, Russo et al.^74^ described positive results by using educational activities developed by organizations that support individuals with DS and conducted by multi-professional teams during the pandemic. Support strategies centered on the individual with DS and their caregivers, as well as healthy eating and physical activity habits, were shown to be fundamental approaches for COVID-19 prevention in the DS population.^74^

In the same direction, Del Carmen et al.^75^ noted that individuals with DS have cognitive traits, such as constancy, tenacity, and a tendency to imitate and repeat behavior, that is interiorized and encourages them to commit to proposed tasks. Therefore, the fact that individuals with DS show easy adherence to behaviors, combined with good actions, may be an essential strategy to disseminate protective factors against SARS-CoV-2 infection. This could be linked to a significant decline in infections in individuals with DS following the first wave when numerous preventative measures had already been disseminated. These concepts cannot be applied to individuals with DS with a severe or profound intellectual deficit or dementia, which may explain why dementia appeared as an essential factor at risk.^75^

### Study limitations

This study has some limitations. Most selected articles have a low level of evidence, and no clinical trials or meta-analyses were found. Furthermore, the review includes publications published until October 14, 2022, and studies published after that were not included.

## CONCLUSIONS

This integrative review allowed us to identify studies that address the behavior of SARS-CoV-2 infection in individuals with DS. This population’s susceptibility to COVID-19 illness is associated with predisposing factors such as previous comorbidities, particularly dementia, immunological dysfunctions, and environmental issues. This may confer a higher risk of infection and an unfavorable clinical course.

The precise pathways involved in the pathophysiology of COVID-19 in individuals with DS are still unclear. Future research developed with different methods—for instance, experimental and clinical studies—may lead to a better understanding of this issue.

Vaccination is currently the most effective strategy to prevent contamination by COVID-19 and its unfavorable outcomes among individuals with DS, as well as in the general population. Maintaining good hygiene by cleaning the hands regularly and thoroughly disinfecting surfaces frequently, especially those often touched, such as door handles, faucets, and phone screens is important. Environmental measures such as avoiding spaces that are closed, crowded, or involve close contact should also be encouraged. To improve ventilation at home or school, it is recommended to bring in as much outdoor air as possible, for example, by opening windows; increasing air filtration in the heating, ventilation, and air conditioning systems by changing filters frequently and using filters that are properly fitted and provide higher filtration; using portable high-efficiency particulate air cleaners; and turning on exhaust fans or using other fans to improve airflow. Appropriate use of masks should still be considered in settings with multiple exposure risks.
